# Identification of Balance Deficits in People with Parkinson Disease; is the Sensory Organization Test Enough?

**DOI:** 10.4172/2329-9096.1000322

**Published:** 2016-01-18

**Authors:** G Gera, DL Freeman, MT Blackinton, FB Horak, L King

**Affiliations:** 1; 2; 3; 4

**Keywords:** Rehabilitation, Parkinson’s disease, Posturography

## Abstract

**Background and Purpose:**

Balance deficits in people with Parkinson’s disease can affect any of the multiple systems encompassing balance control. Thus, identification of the specific deficit is crucial in customizing balance rehabilitation. The sensory organization test, a test of sensory integration for balance control, is sometimes used in isolation to identify balance deficits in people with Parkinson’s disease. More recently, the Mini-Balance Evaluations Systems Test, a clinical scale that tests multiple domains of balance control, has begun to be used to assess balance in patients with Parkinson’s disease. The purpose of our study was to compare the use of Sensory Organization Test and Mini-Balance Evaluations Systems Test in identifying balance deficits in people with Parkinson’s disease.

**Methods:**

45 participants (27M, 18F; 65.2 ± 8.2 years) with idiopathic Parkinson’s disease participated in the cross-sectional study. Balance assessment was performed using the Sensory Organization Test and the Mini-Balance Evaluations Systems Test. People were classified into normal and abnormal balance based on the established cutoff scores (normal balance: Sensory Organization Test >69; Mini-Balance Evaluations Systems Test >73).

**Results:**

More subjects were classified as having abnormal balance with the Mini-Balance Evaluations Systems Test (71% abnormal) than with the Sensory Organization Test (24% abnormal) in our cohort of people with Parkinson’s disease. There were no subjects with a normal Mini-Balance Evaluations Systems Test score but abnormal Sensory Organization Test score. In contrast, there were 21 subjects who had an abnormal Mini-Balance Evaluations Systems Test score but normal Sensory Organization Test scores.

**Discussion and Conclusions:**

Findings from this study suggest that investigation of sensory integration deficits, alone, may not be able to identify all types of balance deficits found in patients with Parkinson’s disease. Thus, a comprehensive approach should be used to test of multiple balance systems to provide customized rehabilitation.

## Introduction

Falls in people with Parkinson’s disease (PD) are multifactorial in origin but postural instability and limitations in gait are a major contributor [1,2]. Limited mobility and falls increase the risk of fractures, poor quality of life and reduced longevity [3,4]. Therefore, fall prevention is an important aspect of healthcare in PD. It is therefore important to recognize balance deficits early in the disease course and to provide customized therapy to the individuals affected by PD. Detection of these balance deficits necessitates use of appropriate clinical assessment tools.

Balance relies upon a complex interaction of multiple physiological systems such as biomechanical constraints, sensory integration, postural responses, cognitive processing, movement strategies etc. [5,6]. Thus, assessment of any one system in isolation may miss critical limitations in balance. Also, focusing balance rehabilitation on only one system underlying balance control may not provide the desired improvements in balance function. Balance deficits in PD are multifactorial in origin, suggesting that many factors can lead to falls in this population [7]. Thus, identification of the specific system deficits is crucial in customizing balance rehabilitation [8].

Despite the advancements in understanding multi-system control of balance, clinical assessment of balance still often focuses only on a single system of balance control. For example, the Sensory Organization Test (SOT) is sometimes used in isolation to identify balance deficits [9–11]. SOT quantifies deficits in the integration of visual, vestibular and somatosensory inputs in maintaining balance by systematically manipulating the three sensory channels during standing balance. The SOT during dynamic posturography (Neurocom/Natus, Inc.) manipulates sensory channels by rotating the visual surround and/or the support surface in proportion to body sway while attempting to stand quietly with eyes open or closed [12]. While people with PD may have deficits in the SOT, it is well established that people with PD also have deficits in other domains, such as gait and postural transitions [8,13–16].

Horak and colleagues developed the clinical balance assessment tool (BESTest) to assess multiple systems underlying balance control [6]. A concise version of the BESTest, i.e. Mini-Balance Evaluations Systems Test (Mini-BESTest) shortened the BESTest to enable evaluation in routine clinical practice [17]. Mini-BESTest tests four systems underlying balance control: anticipatory, reactive, sensory integration, and dynamic balance during gait. The posturography platform used for SOT is expensive and is not routinely available so it is important to determine whether the sensory Integration section of the Mini-BESTest can substitute for SOT in identifying sensory deficits in patients with PD.

The purpose of our study was to determine whether the balance deficits in people with PD identified with the Mini-BESTest and SOT are related to each other. We hypothesized that less people will be identified with abnormal balance with SOT than with the Mini-BESTest. In addition, we hypothesized that the sensory domain of the Mini-BESTest will relate to the SOT.

## Methods

### Subjects

45 participants (27M, 18F; 65.2 ± 8.2 years) with idiopathic PD participated in the cross-sectional study. The data analyzed for the current study pertains to the baseline assessment performed as part of a larger exercise intervention study [18]. Therefore, the number of subjects are based on the larger intervention study. The data was collected at the outpatient balance disorders laboratory at the Oregon Health & Science University (OHSU). Subjects included in the study were diagnosed with idiopathic PD by a movement disorders neurologist. Individuals were excluded from the study if they were not able to walk without assistance, had other neurologic, cardiovascular or orthopedic problems, which could impact mobility or had cognitive deficits that would limit participation in a group exercise program. All participants signed informed consent forms approved by the OHSU Institutional Review Board.

### Protocol

All participants underwent testing for disease severity and deficits in balance using clinical scales when they were in the ON levodopa state. Disease severity was determined by using Unified Parkinsons Disease Rating Scale (UPDRS) III motor section [19] and Hoehn & Yahr (HY) [20]. Disease severity based on the Motor UPDRS was 30.1 ± 14.3, out of a maximum of 108. Disease severity based on the HY was 2.4 ± 0.65. A score of 3 and above indicates postural instability as defined by an abnormal stepping response to a backwards pull on the shoulders. Disease duration for the participants was 5.2 ± 5.0 years. Balance assessment was performed using the Mini-BESTest [17] and the SOT. Fallers were defined as people who had more than one self-reported fall in the last 6 months. Based on this criterion, 5 people were identified as fallers.

### Outcome variables

SOT dynamic posturography: The SOT (SMART Equitest, NeuroCom, Clackamas, OR) was used to characterize the deficits in sensory integration for balance. Participants were tested under 6 sensory conditions (three 20-second trials each), where surface and visual surroundings were systematically modified [12]. The SOT is scored on an interval scale, with the highest possible score of 100 indicating no sway at all. The lowest possible score of 0 indicates that a trial could not be completed. Composite scores derived from the SOT was used for further analyses. The SOT composite score is calculated by a) independently averaging the equilibrium scores for conditions 1 and 2; and b) adding these two scores to the equilibrium scores from each trial of sensory conditions 3, 4, 5, and 6 and dividing that sum by the total number of trials.

Mini-BESTest: Balance assessment was performed using the Mini-BESTest, consisting of 14 items, which are divided into four primary systems underlying balance control [17]. Each item is scored on an ordinal scale from 0–2 (0=severe, 1=moderate, 2=normal), with higher score indicating better performance within the associated balance control system. The highest total score is 28 and indicates the best possible dynamic balance performance. Based on a recent publication on the Mini-BESTest [21], we used the rescaled scores for the Mini-BESTest from 0–28 to a 0–100 with each of the 5 quintile indicating a change in balance disorders level grading from mild to normal, moderate, moderately severe, severe and very severe deficits.

### Data analyses

Data was tested for the normality of the data distribution. Based on the Shapiro-Wilk test, data was not normally distributed (p<0.05). Therefore, non-parametric tests were used for statistical analyses. SPSS software version 22 was used for all the analyses. The level of significance was set as 0.05 for all comparisons. All the subjects included in the analyses were able to complete both the balance assessments, i.e. SOT and Mini-BESTest.

**Figure 1:**
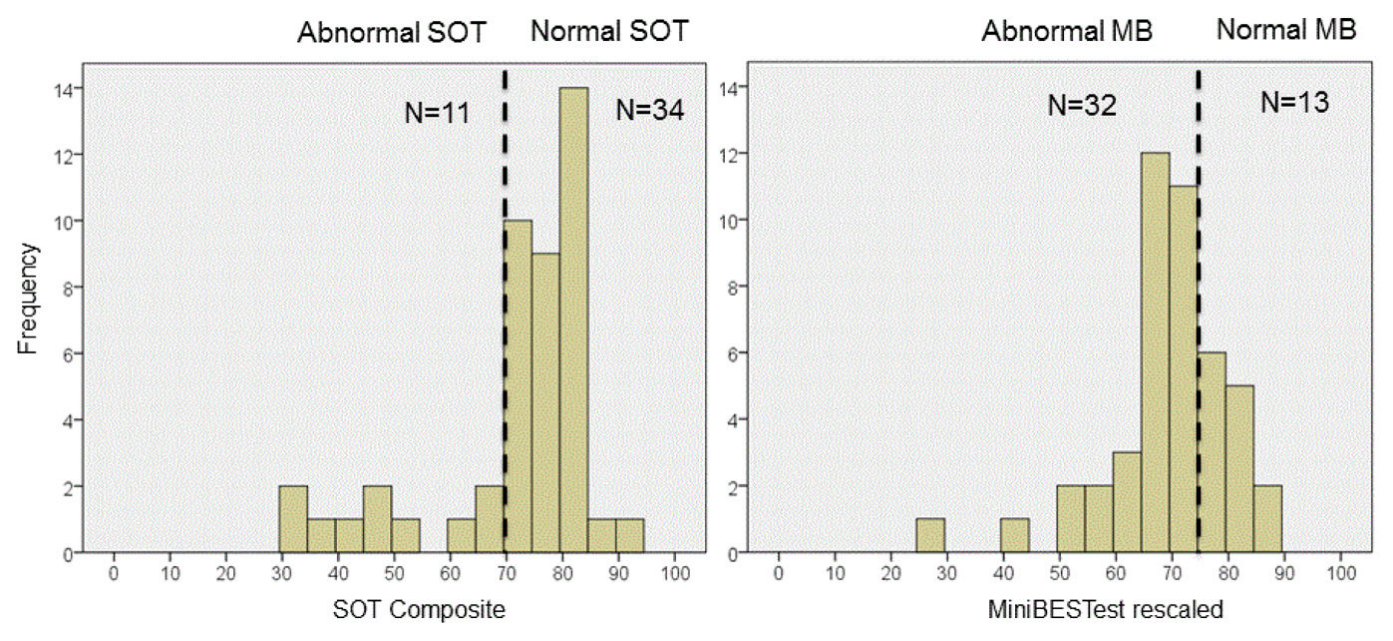
Histograms showing the distribution of scores for SOT and Mini-BESTest scores. Normal (Abnormal) on the Mini-BEST was defined as the score >73 (<76) and for the SOT, Normal (Abnormal) was defined as a score >69 (≤69).

Based on cutoffs of SOT and Mini-BESTest scores (normal balance: SOT>69; Mini-BESTest>73), people were identified with normal and abnormal balance [11,21]. We investigated the frequency distribution to determine the number of people identified with abnormal balance based on two scales (Figure 1). To further determine if the balance deficits identified by both scales were related, we performed correlation analysis between the SOT and Mini-BESTest total and subsections. Due to the limited number of fallers, we qualitatively assessed capacity of SOT and Mini-BESTest to identify fallers.

## Results

More subjects were classified as having abnormal balance with the Mini-BESTest than with the SOT. Figure 1 shows the frequency distribution of the SOT and Mini-BESTest scores. The division of subjects into normal and abnormal balance was different based on the respective, published cutoffs of SOT and Mini-BESTest for identifying patients with balance problems [11,21]. Based on these cutoff scores for the SOT, 11 (24%) people were classified with abnormal balance and 34 (76%) people were labeled as normal at their balance. However, based on the cutoffs of the Mini-BESTest, 32 (71%) participants were classified with abnormal balance and 13 (29%) had normal balance.

**Figure 2:**
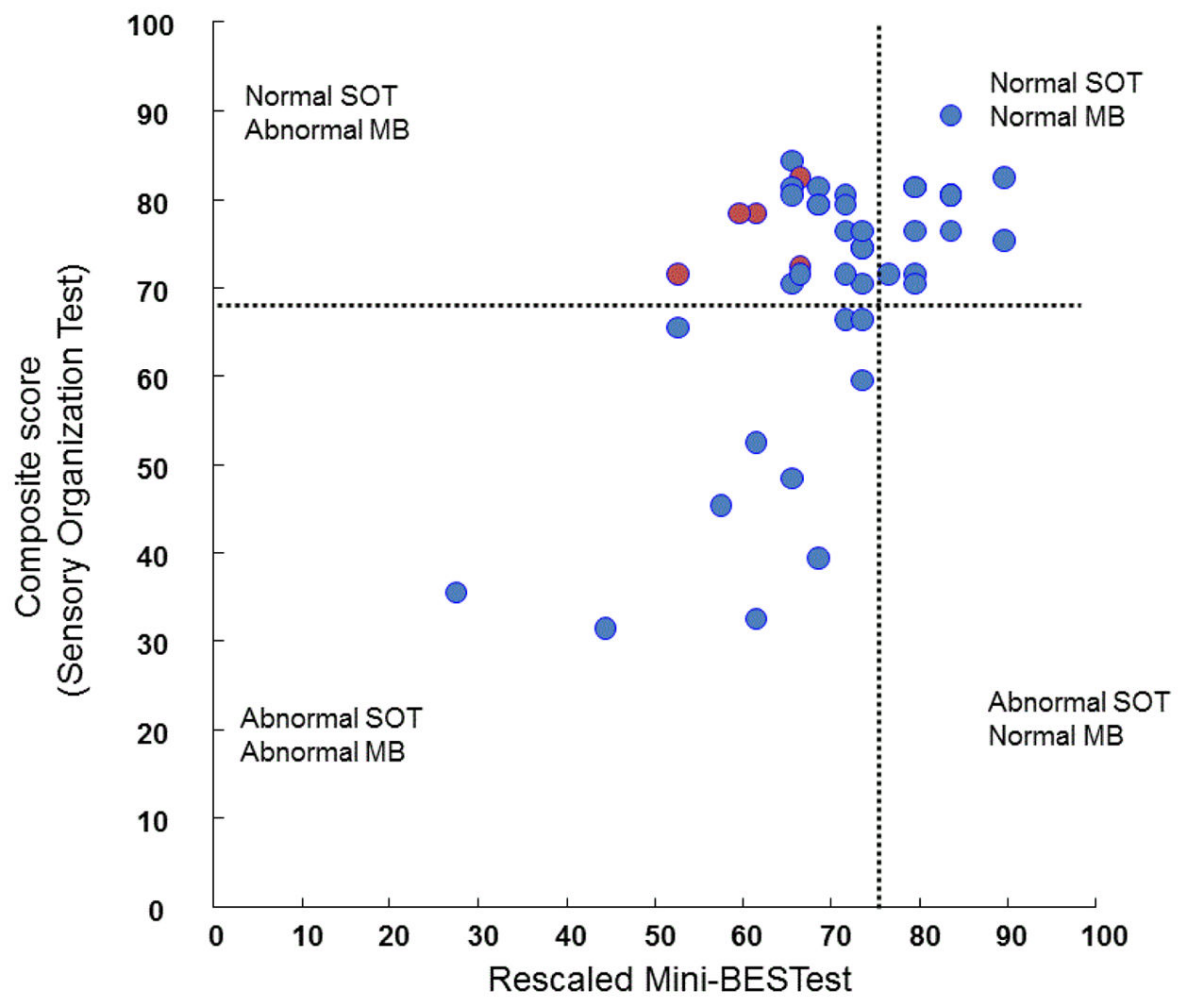
Scatter plot between the composite scores of SOT and the Mini-BESTest (MB) scores. The 4 quadrants represent the subgroups defined on the basis of index of postural instability related to the composite score of the SOT and the total MB scores. Normal (Abnormal) on the Mini-BEST was defined as the score >73 (<76) and for the SOT, Normal (Abnormal) was defined as a score >69 (≤69). Red filled dots represent subjects with 2 falls reported in the last 6 months.

The relationship between the total Mini-BESTest and SOT composite scores was fair, though significant (r=0.41; p<0.01, Figure 2). In addition, the Mini-BESTest scores were related to disease severity as measured by UPDRS (r=−0.7, p<0.01) but the SOT scores were not related to disease severity (r=−0.26, p=0.08).

To further investigate the fair correlation between the two scales and discrepancy in number of subjects with abnormal balance based on Figure 1, we divided subjects into four categories: 1) normal SOT and normal Mini-BESTest, 2) normal SOT and abnormal Mini-BESTest, 3) abnormal SOT and abnormal Mini-BESTest and 4) abnormal SOT and normal Mini-BESTest (Figure 2). As can be seen in Figure 2, there were no subjects with normal Mini-BESTest but abnormal SOT. Thus, none of the subjects with abnormal SOT got overlooked as having normal balance when using the Mini-BESTest. In contrast, there were 21 subjects who had abnormal Mini-BESTest scores but had normal SOT scores (Abnormal MB and Normal SOT).

Correlation analysis between the subdomains of Mini-BESTest and SOT revealed that the SOT composite score was correlated with the sensory domain of the Mini-BESTest for the moderate severity group (H&Y ≥ 3): r=−0.57; p<0.05, but not so for the mild group (H&Y<2): r=−0.02; p=0.9.

Further investigation of the specific balance domains measured by the Mini-BESTest revealed that people with abnormal balance had the worst score in the anticipatory (63.3 ± 3.17 % of maximum score) section, followed by gait (70.0 ± 2.7 % of maximum score), sensory (75.0 ± 3.5 % of maximum score) and reactive (80.8 ± 4.2 % of maximum score) domains. Similarly, the 5 fallers scored worst on the anticipatory section (50% of maximum score on the anticipatory test items, 56% on reactive test items, 62% on gait items and 70% on sensory items). Interestingly, due to the preserved capacity of somatosensory integration, these 5 fallers were not detected with abnormal balance by SOT, but did have abnormal scores on the Mini-BESTest (identified as red dots in Figure 2; the Abnormal MB and Normal SOT group).

## Discussion

Findings from the current study suggest that the SOT, alone, is not sufficient to identify balance deficits in people with PD. The Mini-BESTest identified 47% more people with PD as having balance deficits than the SOT. Furthermore, SOT identified fallers as having normal balance. The Mini-BEST identified deficits in anticipatory postural control, postural responses and gait, as well as sensory integration in people with PD.

Balance deficits in people with PD are multifactorial. SOT has been used to objectively quantify balance deficits in people with PD [9–11,14]. Rossi et al. [10] demonstrated that people with pathological balance (SOT ≤ 69) have deficits in sensory integration of balance. However, Landers et al. [11] showed that the SOT cut-off (68.5) was not sensitive in differentiating people with PD into fallers and non-fallers. Insensitivity of SOT in differentiating people into fallers and non-fallers could be attributed to the fact that the balance deficits in people with PD might be related to domains other than sensory, which can go undetected by SOT [22]. This speculation is corroborated by the findings from the current study that anticipatory balance control and dynamic balance control during gait were more affected than the sensory domain in this cohort of people with PD. Similarly, a prospective cohort study recently showed that there is a worsening of balance in multiple domains of balance control in patients with PD over 6 months of interval [22]. Interestingly, scores on the sensory domain of the Mini-BESTest were the best at the baseline and remained that way at the assessment post one year.

Sensory integration deficits can be identified with the sensory section of the Mini-BESTest. The sensory section of the Mini-BESTest correlated well with the SOT composite score, but this was the case only for patients with moderate severity of PD. Low correlation between SOT and Mini-BESTest for the mild group was related to the fact that only one subject in the mild group had SOT score less than 69. We also found that disease severity (UPDRS) was related to the Mini-BESTest score but not with the SOT. This finding could be attributed to the fact that SOT only measures sensory component whereas the UPDRS evaluates gait, postural responses, bradykinesia, etc. that are also directly and indirectly assessed with the Mini-BESTest.

In the current study, all 5 fallers had normal scores on the SOT but not on the Mini-BESTest. This may be attributed to the fact that the SOT addresses the limitations in sensory integration, whereas Mini-BESTest has other domains that encompass balance control. In addition, the SOT measures standing balance, but most falls occur during dynamic conditions involving turns or postural transitions. Thus, it is important to assess dynamic balance during gait, for example, with the Mini-BESTest.

## Limitations of the study

There were only 5 fallers in the group, so the findings related to balance deficits in fallers are not conclusive. In addition, longer version of the Mini-BESTest, i.e. BESTest includes two more systems, i.e. biomechanical constraints and limits of stability, which might also show deficits in people with PD. Another limitation was related to subjects’ selection since all participants were interested in participating in the exercise study. This potential selection bias may limit the generalizability of the findings.

## Conclusion

Findings from this study suggest that investigation of sensory deficits, alone, may not provide relevant information about all types of balance deficits found in patients with PD. Detection of the deficits in specific balance domains can be used to develop customized rehabilitation strategy [8].
